# Impact of hormonal therapy on the detection of promoter hypermethylation of the detoxifying glutathione-S-transferase P1 gene (*GSTP1*) in prostate cancer

**DOI:** 10.1186/1471-2490-6-15

**Published:** 2006-06-27

**Authors:** Jens Kollermann, Carsten Kempkensteffen, Burkhard Helpap, Mark Schrader, Hans Krause, Markus Muller, Kurt Miller, Martin Schostak

**Affiliations:** 1 Urologische Klinik, Charité – Universitätsmedizin Berlin, Campus Benjamin Franklin, Berlin, Germany; 2 Institut für Pathologie, Universitätsklinikum Eppendorf, Hamburg, Germany; 3 Institut für Pathologie, Hegau-Bodensee-Hochrhein Klinikum, Singen, Germany; 4 Urologische Klinik, Klinikum Ludwigshafen, Germany

## Abstract

**Background:**

In spite of excellent cure rates for prostate cancer patients with favorable tumor characteristics, patients with unfavorable characteristics after radical prostatectomy are still at a significantly increased risk of tumor progression. Early adjuvant hormonal therapy (AHT) has been shown to be of prognostic benefit in these patients. Unfortunately initiation and duration of early AHT in the individual patient is based on statistic data. PSA, as the standard prostate marker is neither able to reliably indicate minimal residual tumor disease in the early postoperative phase, nor can it be used for therapy monitoring due to the suppressive effect of hormonal therapy on PSA production. Promoter hypermethylation of the detoxifying glutathione-S-transferase P1 gene (*GSTP1-HM*) has been shown to be the most common DNA alteration of primary prostatic carcinoma which, when used as a marker, is supposed to be able to overcome some of the disadvantages of PSA. However until now information on the impact of hormonal therapy on the detection of GSTP1-HM is lacking. The purpose of our study was to assess the impact of endocrine therapy on the detection of GSTP1-HM by methylation-specific PCR (MSP) in prostate cancer.

**Methods:**

Paraffin embedded tumor samples from the radical prostatectomy (RP) specimens from 15 patients after hormonal therapy (HT) (mean 8 months) were assessed by MSP. In 8 of the patients the GSTP-1 status of the tumors before HT was assessed on the corresponding initial diagnostic biopsies.

**Results:**

Following HT MSP showed GSTP1-HM in 13/15 of the RP specimens. In two patients analysis of the RP specimens failed to show GSTP1-HM. All initial tumor samples (8/8 biopsy specimens) showed GSTP1-HM, including both patients negative for GSTP1 HM in the corresponding RP specimen.

**Conclusion:**

In most cases hormonal therapy appears to not alter GSTP1 HM detection. However the change from a positive to a negative GSTP1 HM status in a subset of the patients may point to an, at least partial androgen dependency. Further studies on a larger cohort of patients are necessary to assess its frequency and the exact hormonal interactions.

## Background

In spite of a stage migration towards early tumor stages and a decreased prostate cancer mortality rate, attributable to the wide spread use of PSA [1], a significant proportion of patients after putative curative radical prostatectomy still presents with unfavorable tumor characteristics (high Gleason score, positive surgical margins, non organ confined tumors) which are indicative for a high risk of tumor progression [2]. Recent data from the EPC Program study group indicate that early early adjuvant hormonal therapy (AHT) after radical prostatectomy is of prognostic benefit in these patients [3]. Therefore an increase in the number of patients submitted to early AHT has to be expected. The submission of patients to AHT, however, is completely based on statistic data. Biomarkers which are able to indicate the need for early AHT and allow therapy monitoring in the individual patient are needed to avoid overtreatment, unnecessary side effects and costs. Unfortunately in this respect PSA, as the standard prostate marker, is of very limited value. Neither is it able to reliably indicate minimal residual tumor disease in the early postoperative phase, nor can it be used for therapy monitoring due to the suppressive effect of hormonal therapy on PSA production. GSTP1-CpG island hypermethylation (GSTP1-HM) has been shown to be a promising new marker of prostate cancer, which is supposed to be able to overcome some of the disadvantages of PSA. Promoter hypermethylation of the detoxifying glutathione-S-transferase P1 gene (*GSTP1*) is the most common DNA alteration of primary prostatic carcinoma and has already been assessed in prostatic tissue, lymph nodes and bodily fluids of prostate cancer patients by our own group and others [4-8]. However until now information on the impact of hormonal therapy on the detection of GSTP1-HM is lacking. In a pilot study we analyzed tumor tissue from a highly selective cohort of patients before and after long-term neoadjuvant hormonal therapy for the presence of GSTP1-HM by methylation specific PCR (MSP).

## Methods

Representative, paraffin-embedded tumor samples of the radical prostatectomy specimens with a tumor content of at least 50% from 15 patients were studied by MSP. Probe retrieval was done in agreement with the Helsinki Declaration. Institutional review board approval (Ethics committee of the Charité – Universitätsmedizin Berlin) was obtained for this study. All patients signed a consent form approved by the Committee on Human Rights in Research at our institution.

All patients have been submitted to prior complete androgen deprivation therapy until PSA nadir (mean 8 months). In 8 of the patients tumor containing, paraffin embedded prostate biopsies before the initiation of hormonal therapy (HT) were obtained for MSP. In the remainder of the patients biopsy material of the untreated tumors could not be obtained due to tissue loss after routine pathological work up (small tumor foci, loss during the recutting process, immunohistochemistry). Several 5 microm thick recuts were obtained from each paraffin block. One sections of each block was stained with H&E to confirm the presence of prostate cancer in the recuts prior to MSP. The remaining unstained sections were used for DNA extraction as follows: Sections were deparaffinized using 100% xylene followed by 100% ethanol. The pellet was then resuspended in a lysis buffer containing proteinase K. DNA was isolated by the QIAamp DNA Mini Kit (QIAgen, Hilden, Germany). Using the CpGenome DNA Modification Kit (Intergen, Oxford, UK), DNA was modified by bisulfite treatment for the detection of methylated CpG residues, according to the manufacturer's recommendations. *GSTP1 *promoter hypermethylation, i.e. the presence of methylated CpGs in the promoter region was qualitatively assessed by MSP [5]. The target region for MSP lies within the *GSTP1 *promoter. *GSTP1 *promoter sequences were used for the methylated reaction (92-base pair product with blue fluorescence, representing neoplastic DNA; (forward primer: 5'-6FAM-TTC GGG GTG TAG CGG TCG TC-3'; reverse primer: 5'-GCC CCA ATA CTA AAT CAC GAC G-3') and for the unmethylated reaction a 99-base pair product with green fluorescence, representing normal unmethylated DNA was detectable (forward primer: 5'-HEX-GAT GTT TGG GGT GTA GTG GTT GTT-3'; reverse primer: 5'-CCA CCC CAA TAC TAA ATC ACA ACA-3') [7]. MSP (15 minutes at 95°C; 35 cycles at 59°C for 30 seconds; 72°C for 30 seconds; and 95°C for 30 seconds) was performed in a 10-μl reaction volume using HotStar Taq Master Mix (QIAgen, Hilden, Germany). The MSP products from the unmethylated and methylated reactions were analyzed by laser fluorescence on an automated gene sequencer using Gene Scan software, as described elsewhere [5]. In each case with a negative finding MSP was performed twice. Negative (water blank) and positive (DNA from LNCaP cells with a known *GSTP1 *promoter hypermethylation) controls were included in the experiments.

## Results

The relevant clinical data of the patients are shown in table 1. Mean age at the time of diagnosis was 65 years (range 57 – 73). Median initial PSA was 14.4 ng/ml (range 6.4 – 63.7 ng/ml).

MSP showed *GSTP1 *promoter hypermethylation (*GSTP1 *HM) in 13/15 of the radical prostatectomy samples. The majority of the tumors showed moderate regression (regression grade 2). Only two patients showed minor, one showed major regressive tumor changes after HT. In two RP specimens MSP failed to show GSTP1-HM (fig. 2). Both tumors showed moderate regressive change (table 1).

**Figure 1:**
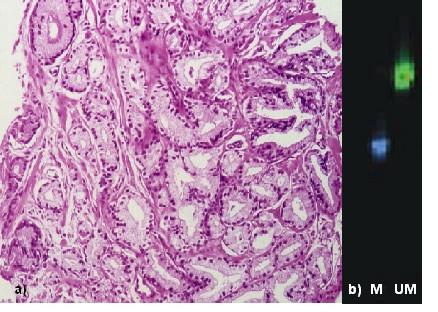
a) Prostate biopsy showing well differentiated adenocarcinoma of the prostate prior to neoadjuvant antiandrogen therapy (H&E staining). b) *GSTP1 *promoter methylation analysis of the tumor displaying both methylated (M, 92-base pairs (bp) product, blue fluorescence; tumor DNA) and unmethylated (UN, 99-bp product, green fluorescence) *GSTP1 *promoter sequences.

**Figure 2:**
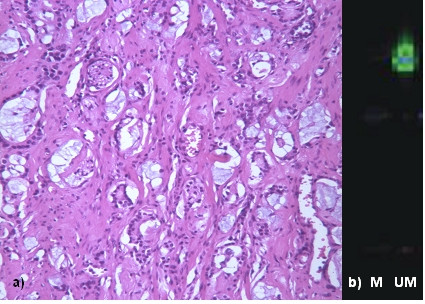
a) Radical prostatectomy specimen of the same patient after NHT showing adenoacarcinoma with moderate regressive changes (H&E staining). b) *GSTP1 *promoter methylation analysis of the tumor displaying only unmethylated (UN, 99-bp product, green fluorescence) *GSTP1 *promoter sequences. Methylated *GSTP1 *promoter sequences (M, 92-base pairs (bp) product, blue fluorescence; tumor DNA) are lacking.

All prostate tumor biopsies obtained prior to HT, showed GSTP1-HM (8/8). Interestingly this includes both patients in whom MSP of the RP specimens failed to show GSTP1-HM (table 1, fig. 1).

## Discussion

Since the knowledge about the close association of GSTP1 CpG island hypermethylation and prostatic carcinogenesis [9], an increasing number of studies evaluated its potential as prostate cancer biomarker. Its feasibility for diagnosis and staging as well as its prognostic value have been intensively assessed in prostate tissue (biopsies and surgical specimens), biopsy washings, ejaculates, blood, urine and lymph nodes by our group and others [4-6,8]. Continuous technical improvements from southern blot analysis to polymerase chain reaction (PCR), which was further improved by the introduction of the methylation specific technique either qualitative or quantitative up to multigene methylation analysis lead to a steady increase of the diagnostic and prognostic power [10-13]. The possibility to detect GSTP1-HM in the blood serum from prostate cancer patients [5,7] and recent reports about the prognostic significance of its detection [14] indicate, that the determination of GSTP1-HM in blood serum may represent a very promising tool for the follow-up of prostate cancer patients. In face of an expected increase in early AHT and the above-mentioned limitations of PSA, the determination of GSTP1-HM in the blood serum appears to be very promising. However, at least to our knowledge, information on the impact of hormonal therapy on its detection is lacking.

In most of the cases analyzed (13/15), MSP was able to detect GSTP1-HM after prolonged neoadjuvant hormonal therapy. However in two patients we failed to demonstrate GSTP1-HM after HT. The presence of GSTP1-HM in the initial biopsies of both patients indicates, that HT may influence the detection of GSTP1-HM in a subset of patients.

In addition to the supposed hormonal interaction other possible explanations for our findings have to be critically discussed.

First of all the lack of GSTP1 HM may be attributable to technical problems (false negative MSP). Although this cannot be excluded completely, several facts argue strongly against this assumption:

1. Prior to DNA analysis the presence of tumor (at least 50% of the sample) in the RP sample was controlled by microscopic assessment of the corresponding HE stained slides (see methods),

2. Both MSP negative samples were analyzed twice,

3. Internal negative (water blank) and positive controls (DNA from LNCaP cells) were always included in the experiments.

4. Unmethylated GSTP1 promoter sequences were seen in both specimens.

Furthermore one has to take into account the possibility that both tumors primarily lacked GSTP1 HM. However the presence of GSTP1 HM in the corresponding tumor biopsies prior to hormonal therapy argues strongly against this assumption.

Due to the general problem to obtain prostate cancer tissue samples before and after hormonal therapy even in cooperation with a specialized center of uropathology (B.H) the number of specimens analyzed in our study admittedly is very small and the results therefore only allow to conclude that androgen ablation therapy appears to influence GSTP1 HM. To assess the true extent further studies on larger cohorts of patients are necessary.

## Conclusion

In most cases hormonal therapy appears to not alter GSTP1 HM detection. However the change from a positive to a negative GSTP1 HM status in a subset of our patients may point to an, at least partial androgen dependency. Further studies on larger cohorts of patients are mandatory to assess its frequency and the exact hormonal interactions.

## Competing interests

The author(s) declare that they have no competing interests.

## Authors' contributions

Authors J.K. and M. Schostak drafted the manuscript. Authors B.H. and K.M. have made substantial contributions to the conception and design of the study. Authors M. Schrader and M.M. revised the manuscript critically. Author CK has made substantial contributions to the acquisition of data. Author HK has made substantial contributions to the conception of the study, analysis and interpretation of data.

## Source of funding

None for all authors

## Pre-publication history

The pre-publication history for this paper can be accessed here:



## Figures and Tables

**Table 1:** Findings in the prostate cancer cohort (n = 15) showing pT-stage, final Gleason score, grade of tumor regression according to Helpap et al [15] in correlation to GSTP1 HM status in the initial prostate biopsy and the corresponding radical prostatectomy specimens after neoadjuvant hormonal therapy.n.m. = no material, pos = positive

**Case**	**pT-stage**	**Final Gleason score**	**RG**	**Biopsy GSTP1**	**RP GSTP1**
1	2	8	1	n.m.	pos
2	2	7	2	n.m.	pos
3	2	7	2	n.m.	pos
4	2	8	2	pos	pos
5	2	6	2	n.m.	pos
6	2	8	2	pos	pos
7	2	7	2	n.m.	pos
8	2	6	2	n.m.	pos
9	2	6	3	pos	pos
10	2	6	2	n.m.	pos
11	2	6	2	pos	pos
12	2	7	2	pos	pos
13	2	7	2	pos	neg
14	2	6	2	pos	neg
15	2	7	1	pos	pos
